# A Microtube-Based Wearable Closed-Loop Minisystem for Diabetes Management

**DOI:** 10.34133/2022/9870637

**Published:** 2022-10-25

**Authors:** Yiqun Liu, Qi Yu, Xiaojin Luo, Le Ye, Li Yang, Yue Cui

**Affiliations:** ^1^School of Materials Science and Engineering, Peking University, China; ^2^First Hospital Interdisciplinary Research Center, Peking University, Beijing, P. R., China; ^3^Renal Division, Peking University First Hospital, China; ^4^Peking University Institute of Nephrology, China; ^5^Key Laboratory of Renal Disease, Ministry of Health of China, China; ^6^Key Laboratory of Chronic Kidney Disease Prevention and Treatment (Peking University), Ministry of Education, Beijing, P. R., China; ^7^School of Integrated Circuits, Peking University, Beijing, P. R., China

## Abstract

Diabetes is a chronic metabolic disease with a high blood glucose level, leading to both seriously acute and chronic complications. The closed-loop system is an ideal system for diabetes management. However, the large size and high cost of the commercial systems restrict their widespread uses. Here, we present for the first time a microtube-based wearable closed-loop minisystem for diabetes management. The closed-loop minisystem includes a biosensing device, an electroosmotic micropump, and a printed circuit board (PCB) with an algorithm. The microtube-based sensing device coated on the outer surface of the microtube is inserted into subcutaneous tissue for detecting interstitial glucose; the current signal for sensing glucose is processed by the PCB to power the electroosmotic micropump intelligently for the delivery of insulin into the subcutaneous tissue via the microtube channel. The closed-loop minisystem worn on a diabetic SD rat can successfully maintain its blood glucose level within a safe level. It is expected that this new closed-loop paradigm could open up new prospects for clinical diabetes management.

## 1. Introduction

Diabetes, as a global chronic disease among millions of people [1, 2], is caused by the inability to produce insulin because of pancreatic dysfunction (type I diabetes) [3] or caused by the malfunction of cells to use insulin (type II diabetes) [4]. Diabetes management is highly important for diabetes patients, since a lack of proper management could result in serious complications, such as heart disease, stroke, kidney failure, blindness, and nerve damage [5–7]. The key for a desired management is to control the blood glucose at a normal level, usually in the range of less than 7.0 mM [8].

Diabetes patients usually need three to four blood glucose concentration samples per day obtained by the patient by using fingerstick glucose meters, which is painful and inconvenient [9]. When blood glucose is higher than a normal level, patients would take oral drugs (such as insulin secretagogues [10], noninsulin secretagogues [11], DPP-4 inhibitors [12], and SLGT-2 inhibitors [13]) or subcutaneously inject insulin. These ways are inconvenient for the patients and always result in a poor control of diabetes. To improve the user experience and the working performance, novel wearable biosensors and drug delivery devices have been studied, such as the flexible self-sustainable sweat sensors [14], the integrated watch for continuous glucose monitoring [15], and the multimicrochannel microneedle for intracellular drug delivery [16]. Recently, the closed-loop devices have attracted great interest due to their intelligent function for automatic monitoring blood glucose and injecting insulin, such as the system developed by Medtronic Inc. (Northridge, CA) [17]. The device normally includes a continuous glucose meter and a pump in separated parts, and the pumping amount of insulin is adjusted by the glucose level, which is controlled by an electronic chip with algorithm. Therefore, the closed loop is usually very large in size, and it normally has a dimension of 5 × 9 × 2 cm.

The intradermal route is feasible for sensing glucose and delivering insulin [18–21]. Several studies have been performed to develop the closed-loop devices towards the dermis layer, such as a nanosensor array on microneedle elements for monitoring glucose levels and delivering insulin [22], the wearable patch based on a sweat biosensor and polymeric thermoresponsive microneedles [23], and a system based on mesoporous microneedles and iontophoresis technology [24]. We have recently shown a microneedle sensing device with an electroosmotic micropump for a closed-loop diabetes management [25] as well. However, the microneedles can only reach the dermis layer, and these devices suffer from some key disadvantages, such as the unreliability of sensing components, the imprecise control of the insulin release rate, and the leakage of insulin due to the small height of the microneedles. In addition, the capillaries in the dermis are not abundant which may limit the exchange rate of the glucose molecules between cells and blood vessels to result in a time delay for glucose sensing.

Subcutaneous tissue has more capillaries to result in an enhanced exchange rate of glucose molecules between cells and blood vessels. The time lag between glucose fluctuation in subcutaneous tissue and glucose fluctuation in blood is shorter than that in the dermis layer. In addition, insulin can be absorbed faster in the subcutaneous tissue than that in the dermis. There is no insulin accumulation in the subcutaneous tissue or insulin leakage out of the skin.

A microtube is usually made of a flexible polymer [26, 27], or a metal [28]. Compared with a metal, a polymeric microtube has a high biocompatibility, causes less pain, and can control the implanting angle conveniently [29, 30]. A microtube with a length of several millimeters could be implanted into subcutaneous tissue, usually for delivering a drug, such as insulin [31], tenofovir alafenamide [32, 33], dexamethasone [34], and estradiol-17 beta [35].

In this work, we show for the first time a flexible, biocompatible microtube-based closed-loop minisystem for diabetes management. The outer surface at one end of the microtube is coated with a biosensing device to detect the glucose levels in subcutaneous interstitial fluid continuously. The other end of the microtube is integrated with an electroosmotic micropump that can provide a precise insulin delivery into subcutaneous tissue through the microchannel of the microtube. A printed circuit board (PCB) is connected to both the sensor and the micropump, to power the sensor, detect the sensing signal, and drive the micropump to deliver the insulin according to the sensing signal from subcutaneous glucose. The microtube enables an effective injection of insulin into subcutaneous tissue without a leakage or a low adsorption. The biosensing device on the outer device with a length along the entire dermis and subcutaneous tissue enables an effective sensing of glucose. The closed-loop device is wearable, small, and cost-effective.

## 2. Results and Discussion

### 2.1. Overall Device Principle and Components of the Closed-Loop System

**Figure 1:**
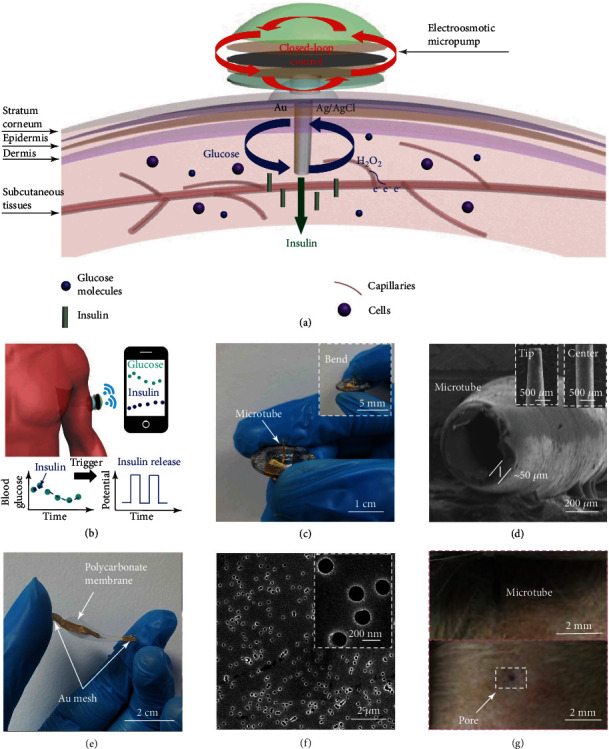
Schematic illustrations and images of the closed-loop system with a glucose microtube biosensor and an electroosmotic micropump. (a) Scheme of the system and the working process in the subcutaneous tissue. (b) Illustrations of the system applied to the human body. (c) A camera image of the flexible microtube biosensor. (d) SEM images of the microtube. (e) Camera image of the key components of the electroosmotic micropump. (f) SEM images of the commercial nanoporous polycarbonate membrane. (g) Camera images of the insertion process of the flexible microtube and the pore left on the skin.

As illustrated in Figure 1(a), the system included a glucose biosensor with two electrodes deposited on the opposite sidewalls of the microtube, an electroosmotic insulin micropump, and a flexible catheter to connect them. The flexible microtube with a diameter of 400 *μ*m and a length of 6 mm was inserted into the subcutaneous tissue transdermally, passing through the epidermis (75–150 *μ*m) and dermis layer (1–4 mm) [36]. The Au and Ag/AgCl electrode was able to provide an electrochemical sensing of glucose. This transdermal sensing approach for glucose has been proven to be feasible, since many glucose molecules were transferred from capillaries to interstitial fluid when the blood glucose level changes rapidly. The nanoporous polycarbonate membrane was the core of the micropump to achieve an electroosmotic flow. Two stainless-steel meshes with an Au-layer deposition were adhered to both sides of the membrane as the anode and cathode of the micropump. Before placing the system on a skin, the skin surface at the penetration position was disinfected by ethanol without needing a local anesthesia. The system was then worn on the skin (such as abdomen and upper arm) and penetrate the skin surface into the subcutaneous tissue.

Figure 1(b) illustrates the working principle of the system. The microtube was inserted into the arm of the human body to perform a glucose sensing and an insulin release. Glucose was catalyzed by glucose oxidase (GOD) to produce H_2_O_2_ that was further detected by the sensing electrode to result in a current response. A high blood glucose level triggered the PCB automatically to apply a constant potential on the electroosmotic micropump for releasing insulin through the hollow microtube. With the injection of insulin into subcutaneous tissue, the blood glucose level gradually declined to a normal range. The system could be further connected with a wireless unit for transmitting the blood glucose data to a portable device (e.g., smartphone), and users could know the fluctuations of blood glucose conveniently.

Figure 1(c) shows the camera images of the microtube biosensor. The microtube was flexible and can penetrate into the skin with the assistance of a stainless-steel puncture guide needle core. Figure 1(d) shows the SEM images of the flexible microtube. The tip of the microtube was in a trapezoid shape, and the center of the microtube was in a cylinder shape. The microtube was 6 mm in length and 0.4 mm in diameter with a sidewall thickness of about 50 *μ*m. Figure 1(e) exhibits a photograph of a polycarbonate electroosmotic micropump with two Au-deposited stainless-steel meshes adhered on its two sides as the electrodes. The diameter of the circular polycarbonate membrane was 2.5 cm with a thickness ranging from 7 to 22 *μ*m. The rectangular mesh was 1 cm in width and 2.5 cm in length with a thickness of about 100 *μ*m. A 3D-printed hemispherical-shaped drug reservoir (2 cm in diameter) was adhered with the micropump for storing the insulin solution. There were multiple pores (200 nm in diameter) on the surface of the polycarbonate membrane to achieve an electroosmotic flow (Figure 1(f)).

The entire system can be very small and wearable with a centimeter size (Figure S1). The microtube can be inserted into the rat skin successfully (Figure 1(g)). After removing the system, there was no bleeding and severe traumatic wound in the skin. Therefore, there was no serious injury to the skin in using the system.

A printed circuit board (PCB) was used to control the sensing, pumping, and feedback processes for the biosensor and micropump (Figure S2). The PCB can provide the constant potentials for the biosensor (0.1–0.3 V) and the micropump (0–10 V), receive data from the biosensor, transmit the data to the computer, and control the micropump for the insulin release. From the schematic diagram and circuit diagram of the PCB for the biosensor and the micropump (Figures S2 and S3), the current measured by the biosensor was processed by the PCB that determined whether the blood glucose level was higher than the critical value. If the blood glucose was higher, it would drive the micropump to release the insulin solution. After a short period of the insulin release, the PCB would drive the biosensor to perform a glucose sensing again. The sensing and insulin delivering processes would be repeated alternatively until the blood glucose level reached the normal level. The design of this PCB was similar to the one we reported previously [25].

### 2.2. Construction of Biosensor for Detecting H_2_O_2_

**Figure 2:**
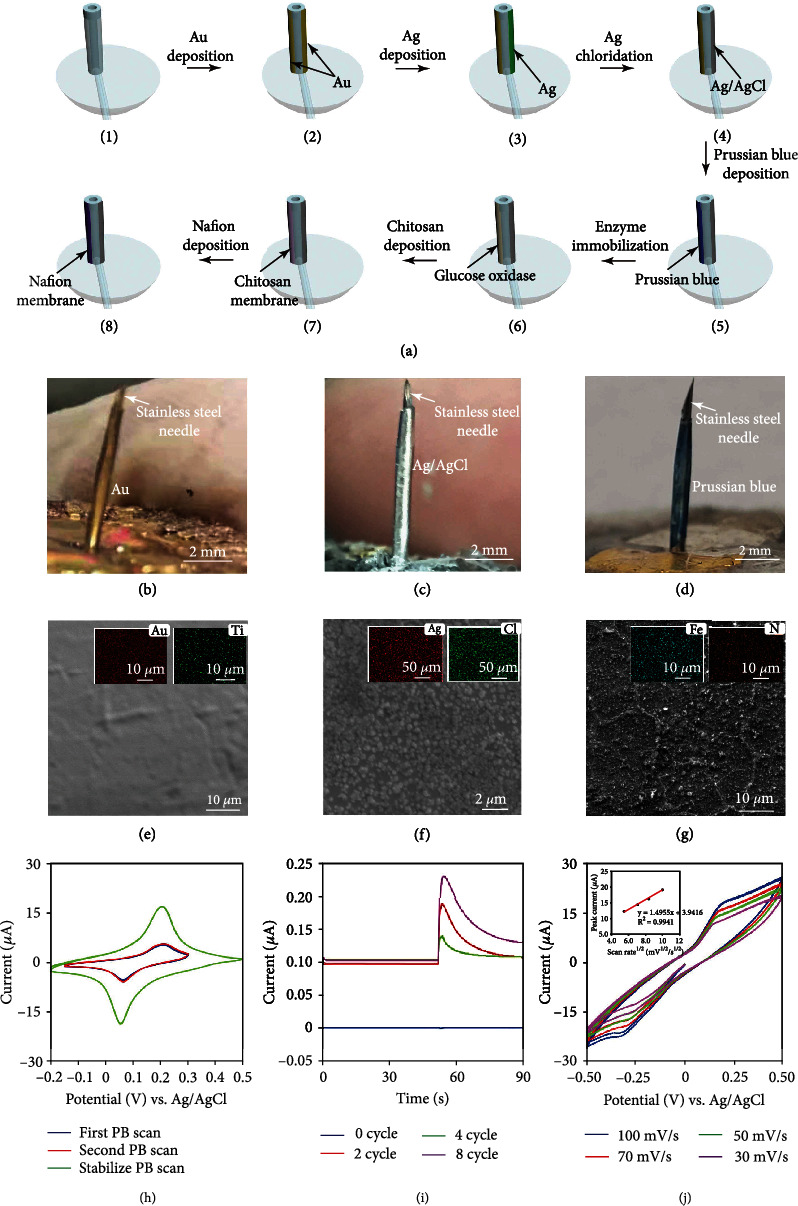
Fabrication process and electrochemical characterization of the microtube biosensor. (a) Fabrication process of the microtube biosensor. (b) Camera image of the Au electrode on the sidewall of the microtube. (c) Camera image of the Ag/AgCl electrode on the side wall of the microtube. (d) Camera image of the Prussian blue layer on the Au electrode. (e) SEM image and EDS analysis of the bare Au electrode. (f) SEM image and EDS analysis of the Ag/AgCl electrode. (g) SEM image and EDS analysis of the Au electrode after the Prussian blue deposition. (h) CV curves for Prussian blue electrodeposition and stabilization on the surface of the Au electrode. (i) Response of the biosensor for detecting 4 mM H_2_O_2_ with different Prussian blue layer thickness. (j) CV curves of the biosensor for detecting 4 mM H_2_O_2_ at different scan rates.

Figure 2(a) illustrates the fabrication process of the biosensor. Two opposite sidewalls of the microtube were deposited with Ti/Au as the working electrode and a base layer for the Ag electrode (1-2). A solid-state Ag/AgCl was designed for being the counter/reference electrode, and it was formed by an Ag layer deposition and its chlorination (3-4). A thin-film Prussian blue (PB) layer was then electrodeposited on the Au working electrode (5), as a mediator for H_2_O_2_ to transfer the electrons to the working electrode. It can ensure the biosensor being operated at a low potential (0.1 V), extend the detection range, and increase the sensitivity. GOD was then immobilized on the Au electrode to catalyze the glucose molecules to generate H_2_O_2_ for sensing (6). The biocompatible chitosan/Nafion membrane was then deposited as the encapsulation matrix for GOD to protect the enzyme from leakage, control glucose diffusion, eliminate interferences, and enhance the biocompatibility of the device (7-8).

As shown in Figures 2(b) and 2(c), each electrode had a width of 0.4 mm and a height of 6 mm, and two electrodes were successfully fabricated on the sidewall of the microtube. The Au electrode was in a yellow color (Figure 2(b)), the Ag/AgCl electrode was formed by chloridizing the Ag layer and in a gray color (Figure 2(c)). After the deposition of PB, the working electrode showed a slight blue color (Figure 2(d)). A stainless-steel needle that was longer than the microtube would guide the microtube to be inserted into the skin. Although the insertion with a stainless-steel guide needle may cause a little pain to the users, the microtube was soft, flexible, and very thin (only 400 *μ*m in diameter) that would minimize the pain feeling after the insertion. It has been demonstrated that the pain was almost the same when using a microneedle and a subcutaneous microtube for the insulin infusion [37]. A variety of microtubes with different dimensions and shapes can be used, and a 400 *μ*m-diameter microtube can also be replaced by a microtube with a smaller diameter [38].

The characterizations by SEM and EDS was performed on a bare Au electrode, an Ag/AgCl electrode, a PB-deposited Au electrode, and a GOD and Nafion-deposited electrode, as can be seen in Figures 2(e)to 2(g) (Figure S4 to Figure S5). The results indicate that Au and Ti elements were distributed on the sidewall of the microtube evenly. Figure 2(f) and Figure S5b also indicate that Ag and Cl elements were also evenly distributed on the Ag/AgCl electrode. Compared to a bare Au electrode, the N and Fe elements were distributed well on the surface of the Au electrode, demonstrating the successful deposition of PB (Fe_4_[Fe(CN)_6_]_3_) on the Au electrode (Figure 2(g) and Figure S5c). The Nafion ((C_7_HF_13_O_5_S·C_2_F_4_)x) membrane was the outmost layer of the Au electrode; the EDS mapping indicates that the S and F elements were also distributed on the Au electrode evenly (Figure S4 and Figure S5d). There were some cracks in the Nafion membrane that may enhance the diffusion of O_2_ and glucose molecules into the enzyme layer.

Figure 2(h) shows the cyclic voltammograms (CV) of the PB-deposited Au electrode in a 0.1 M KCl/HCl solution. A CV scanning was performed on the sensor to deposit PB with 4 cycles each time for twice. The CV performances were stable in the two repeated scanning experiments for depositing PB. After the PB modification, the sensor was immersed into a KCl/HCl solution for a CV scanning from −0.2 V to 0.5 V to stabilize the PB layer. Figure 2(i) shows the current responses of the biosensors with different thicknesses of the PB layers to 4 mM H_2_O_2_. The sensing response with a PB deposition for 8 cycles was faster and larger that was chosen for the further experiments. After the PB deposition, the electron transfer impedance of the electrode declined sharply (Figure S6). Figure 2(j) shows the CV curves of the sensor with 4 mM H_2_O_2_ in PBS. The peak currents of the CV curves were proportional to the square root of the scanning rates, indicating a diffusion-controlled process for the H_2_O_2_ sensing [39–41]. The current values were variable at different potentials due to the different reduction and oxidation rates of H_2_O_2_. A 0.1 V potential was chosen for the further amperometric experiments due to the clear signal response in the CV curves. Figure S7 displays the current response of the sensor for detecting H_2_O_2_ in PBS and the calibration curve between the sensing signal and the H_2_O_2_ concentrations. A linear detection range was clearly obtained from 0.8 mM to 85 mM with a sensitivity of 0.0190 ± 0.0005 *μ*A/mM (*n* = 3). The detection limit was calculated to be 13.78 *μ*M (signal to noise was 3). H_2_O_2_ is the product of glucose molecules oxidized by the GOD, and the successful detection of H_2_O_2_ indicates the possibility to measure glucose accurately.

### 2.3. Electrochemical Sensing of Glucose with the Microtube Sensor

**Figure 3:**
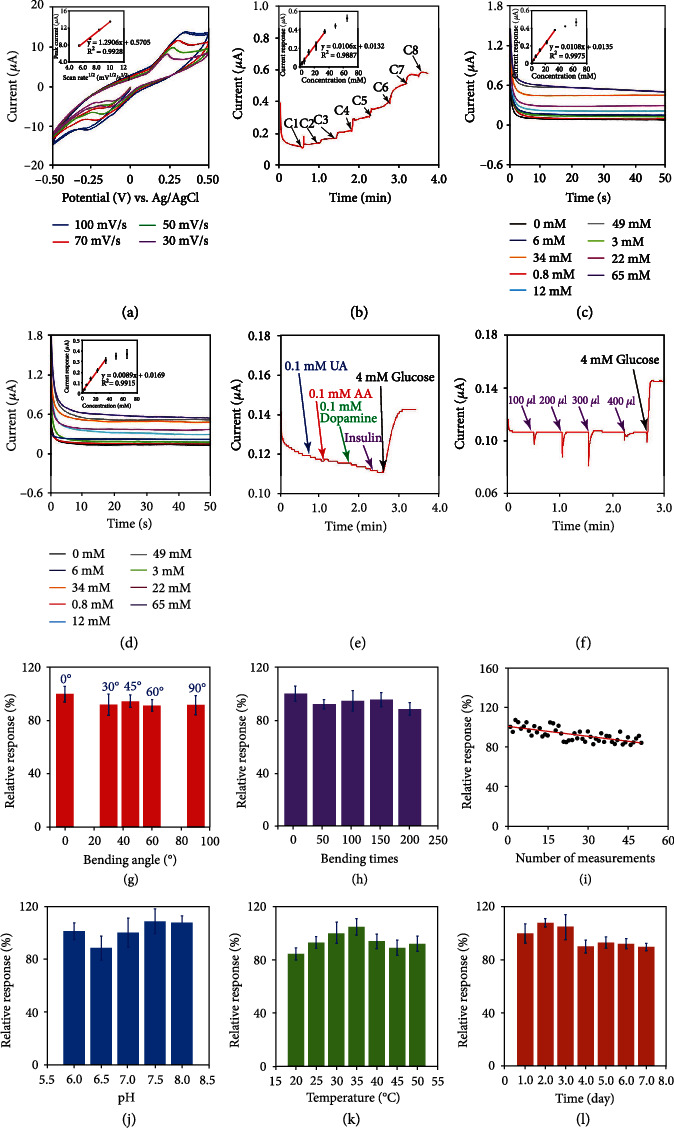
Characterization of the microtube biosensor for sensing glucose. (a) CV curves of the sensor for detecting 4 mM glucose at different scan rates. (b) Current-verses-time response and calibration curve upon the additions of different concentrations of glucose in PBS. C1: 0.8 mM, C2: 2.2 mM, C3: 3 mM, C4: 6 mM, C5: 10 mM, C6: 12 mM, C7: 15 mM, C8: 16 mM (*n* = 3). (c) Current baseline response and calibration curve to different concentrations of glucose in PBS (*n* = 3). (d) Current-verses-time and calibration curve in different glucose concentrations of simulated interstitial fluid (*n* = 3). (e) Amperometric *i* − *t* result of selective response to glucose and different interfering substances (UA: uric acid, AA: ascorbic acid). (f) Amperometric *i* − *t* result of selective response to glucose and different volumes of insulin solution (10 U/ml). (g) Relative response of the biosensor in detecting glucose with different bending angles (*n* = 3). (h) Relative response of the biosensor in detecting glucose with different bending times (each time bending was ~45°, *n* = 3). (i) Repeatability of the biosensor for continuous 50 times measurements of the glucose. (j) The pH stability of the biosensor over 6 to 8 (*n* = 3). (k) The temperature stability of the biosensor over 20 to 50°C (*n* = 3). (l) The storage stability of the biosensor over 7 days (*n* = 3).

Figure 3(a) shows the CV curves of the sensor to 4 mM glucose in PBS with a potential range from −0.5 V to 0.5 V. By changing the potentials, the current values varied, and a linear relationship can be seen between the peak current and scanning rate, indicating a diffusion-controlled process for sensing glucose as well. Figure 3(b) shows the sensing responses to a series of glucose additions in PBS. The biosensor was able to detect a wide glucose concentration range (0.8–34 mM) with a sensitivity of 0.0106 ± 0.00045 *μ*A/mM (*n* = 3). These concentrations could cover the entire blood glucose levels in the diabetic patients as well as the normal persons. Figure 3(c) shows the current baseline response of the sensing device to glucose in PBS. Its performance was similar to that in Figure 3(b) with a sensitivity of 0.0108 ± 0.00025 *μ*A/mM and a linear detection range of 0.8–34 mM (*n* = 3). Figure 3(d) illustrates the currents to different glucose concentrations in simulated interstitial fluid and the calibration curve for the sensor. The calibration curve exhibited a slope of 0.0089 ± 0.00076 *μ*A/mM and a 0.8–34 mM linear range (*n* = 3).

Figure 3(e) shows the selectivity of the sensing device. The selectivity of the biosensor is essential to achieving a high accuracy in detecting interstitial glucose, since many electroactive substances could exert the interfering effects on the glucose sensing. The results showed that the biosensor was more selective to glucose than other electroactive interferences, including uric acid, ascorbic acid, dopamine, and insulin. Figure 3(f) shows the current-versus-time curve of the microtube sensing device after adding different volumes of insulin (10 U/*μ*l) and glucose (4 mM) in PBS. Due to a continuous injection of insulin, the delivered insulin may accumulate around the sensor and affect its accuracy. These results demonstrate that different volumes of insulin almost had no effect on the sensing response to glucose.

In addition, the sensing performance was almost unaffected upon applying different bending angles and bending times on the microtube due to its great flexibility, as can be seen Figures 3(g) and 3(h). The relative response after the microtube being bent for 90°once was 91.69%, and it was 88.67% with a 200 times' bending at 45°. The operational, pH, temperature, and storage stabilities were evaluated, as shown in Figures 3(i)to 3(l). The sensor exhibited the excellent performances (above 80% of the initial response) for over fifty continuous measurements, for sensing at different temperatures, for sensing at different pH values, and for over 7 days. The relative responses of the biosensors at pH 6.5, at 20°C, and at the 7th day were 88.31%, 84.43%, and 84.78%, respectively. All these results demonstrate that the biosensor is sensitive and stable for detecting glucose under different circumstances.

### 2.4. Characterization of the Electroosmotic Micropump

**Figure 4:**
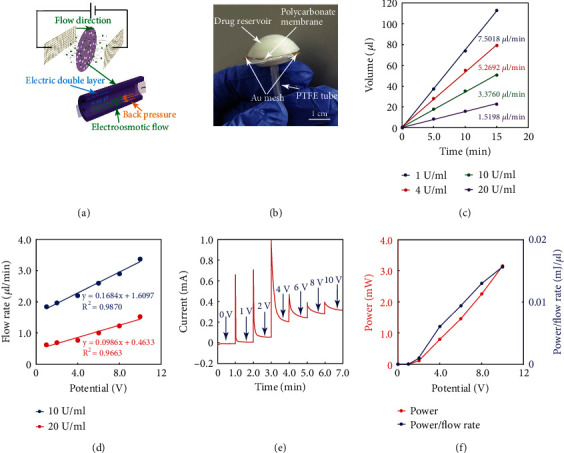
Characterizations of the integrated electroosmotic micropump. (a) Schematic illustration of the working principle of the electroosmotic micropump. (b) A camera image of an electroosmotic micropump with a connection to a PTFE tube (that was further connected to a microtube). (c) The flow rate of the electroosmotic micropump for releasing different concentrations of fast-acting insulin aspart injection solution under a 10 V DC voltage. (d) The flow rate change of the electroosmotic micropump under different DC voltages for pumping different concentrations (10 U/ml and 20 U/ml) of insulin injection solution. (e) The current change of the electroosmotic micropump under different DC voltages for pumping insulin injection solution (10 U/ml). (f) The power and power/flow rate needed of the micropump under different voltages.

Figure 4(a) shows the working principle of an electroosmotic micropump for insulin. The nanopore polycarbonate membrane was the key component of the micropump. Two stainless-steel meshes after the Au deposition was attached with the membrane as electrodes, and a constant DC voltage was applied to electrodes. During its working process, the surface charges from the ionizable groups or a strong adsorption of the charged species were accumulated at walls of the pores. Therefore, the oppositely charged ions from the electrolyte were attracted by these charges to form an electric double layer. Due to a thermal diffusion, a diffusion layer with a concentration gradient of the oppositely charged ions was formed near the walls. By applying an electrical field in the axial direction, the electric force would drive the net charges in the diffusion layer towards the oppositely charged electrode, dragging the solvent along to produce an electroosmotic flow. Figure 4(b) shows the camera image of a micropump. A 3D-printed button shape chamber was adhered to the top of the membrane as the insulin reservoir, and the micropump was connected to a microtube via a medical-grade PTFE hollow tube (4 mm in diameter and ~2 cm in length) in between. The micropump was connected to a PCB, which provided a constant voltage with a range of 0 to 10 V to power the micropump. The PCB was further connected to a computer through a USB cable to transmit the current and voltage data.

Figure 4(c) shows the rate of the electroosmotic micropump at a DC voltage of 10 V for pumping different concentrations of insulin. With an increasing insulin concentration from 1 U/ml to 20 U/ml, the flow rate gradually decreased from 7.5018 *μ*l/min to 1.5198 *μ*l/min, probably due to the elevated viscosity from a high concentration of insulin. Figure 4(d) shows the change of flow rates at different DC voltages for pumping 10 U/ml and 20 U/ml insulin solutions. The flow rate was linearly increased from 1.8393 *μ*l/min (10 U/ml) and 0.6189 *μ*l/min (20 U/ml) to 3.376 *μ*l/min (10 U/ml) and 1.5198 *μ*l/min (20 U/ml) when the potential was increased from 1 V to 10 V. Users could further change the rate of insulin release by adjusting the DC voltage applied to the micropump. Figure 4(e) shows the current-versus-time curve at different potentials for releasing insulin solution (10 U/ml). A larger current was obtained at a higher potential, and the current at each voltage was relatively stable during the measuring time (60 s). Figure 4(f) exhibits the power and power/flow rate for micropump at different potentials. The power needed by the micropump was very low (3.16 mW at 10 V) for delivering insulin (10 U/ml) at a flow rate of 3.376 *μ*l/min. In addition, the ratio of power/flow rate increased with an increasing potential, and this indicates that the micropump was more efficient at a lower potential. All these results indicate that the micropump exhibited an excellent performance for delivering insulin.

### 2.5. In Vivo Performance of the Closed-Loop System

**Figure 5:**
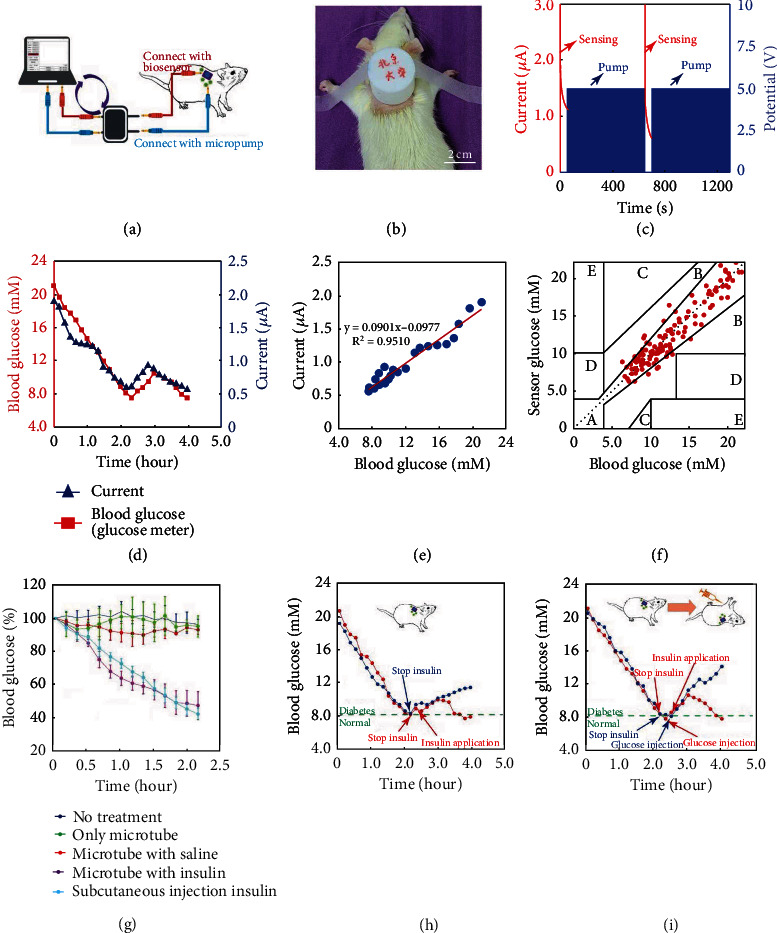
The control of the blood glucose level in diabetic rats with the microtube-based closed-loop system. (a) Schematic illustration of the system applied to an SD rat. (b) A camera image of the SD rat with the closed-loop system. (c) Working operation of the system for the closed-loop management. (d) The blood glucose levels measured by a commercial blood glucose meter and the microtube biosensor. (e) Calibration curve of the microtube biosensor for monitoring blood glucose. (f) Clark error grid of the blood glucose level measured by the biosensor and the blood glucose meter. (g) The change trend of the rat's blood glucose concentration (%) under different conditions. (h) Comparison of the devices with and without the closed-loop function for controlling blood glucose. (i) Comparison of the devices with and without the closed-loop function for controlling blood glucose during a glucose intake.

Before conducting the closed-loop study, normal Sprague Dawley (SD) rats were induced to be diabetic rats by injecting STZ according to the protocol [42]. The closed-loop system was applied on the back skin of a diabetic rat. The microtube sensing device and the electroosmotic micropump were connected to a PCB that exchanged data with a PC (Figure 5(a)). Figure 5(b) shows a camera image of the system applied to an SD rat. The back skin of the rat was selected as the insertion site of the system, and after removing hairs, the microtube was plunged into the skin transdermally. The microtube device was successfully inserted into the rat's skin that was demonstrated from a hematoxylin and eosin-stained pierced skin section (Figure S8a). After applying a 20-gram object on the skin, the rat's skin was almost not changed (Figure S8b). The results indicate that due to its flexibility and softness, the microtube device would not cause serious tissue damage in case of a fall of a wearer. From the camera images of the rat's skin after applying a microtube device for 5 days (Figure S9), there was no obvious skin reaction, such as serious erythema and edema, demonstrating an excellent biocompatibility and safety of the microtube device. After removing the microtube device, the more pores left on the skin disappeared in about four days (Figure S10).

A two-step mode was adopted to achieve a closed-loop management, as illustrated in Figure 5(c). The red color is the current-versus-time (*i* − *t*) curve for sensing glucose (the left line corresponded to 13.1 mM blood glucose, and the right line corresponds to the 8.2 mM). After measuring glucose for 50 s, the final current value of the *i* − *t* curve was recorded and sent to the PCB to decide if the blood glucose level reached the critical value (8.3 mM). If the value was higher than the critical value (8.3 mM), a 5 V potential was applied to the micropump to inject insulin (10 U/ml) for 600 s. The sensing and pumping steps were repeated alternatively until the blood glucose was back to the normal value. After that, the insulin release was stopped and only the glucose sensing process was performed. When the pump was stopped, a 0.1 V potential was provided to the pump to prevent the loss of its flow rate caused by the repeated on/off micropump.

For the automatic closed-loop management of diabetes, the first step is to obtain the relationship between the current measured by the biosensor and the true blood glucose level. As shown in Figure 5(d), the current change from the microtube biosensor correlated well with the blood glucose level measured by a commercial glucometer, and a linear relationship was seen with a sensitivity of 0.0901 *μ*A/mM (Figure 5(e)). All 135 points from six different rats were located in the safe error zone (A and B) of the Clark error grid (Figure 5(f)). The error of the biosensor was from 0.094% to 25.33%, The mean absolute relative difference (MARD) value of the |relative error| between these two detection approaches was 9.341% ± 6.271%, and 75% of points were lower than 14.241% (Figure S11). The results fulfilled the criteria for the accuracy in glucose sensing [43]. All these results indicate that the biosensor could measure the blood glucose level accurately.

Figure 5(g) shows the changes in the blood glucose levels with no treatment, a microtube injection, a saline delivery, an insulin delivery, and the one-time subcutaneous injection of insulin. No decrease in the blood glucose levels can be seen with no treatment, a microtube injection, or a saline delivery. When the microtube was applied to a rat and insulin was released by a pump, the blood glucose level was lowered to 45% of its initial value in two hours. In addition, the hypoglycemic ability of the system was similar to the one-time subcutaneous injection of the same amount of insulin in 130 min (the amount of insulin was calculated according to the flow rate of the micropump).

Figure 5(h) shows the closed-loop management of diabetes without a glucose intake. The blood glucose of the first rat (blue line) was declined dramatically in about two hours. At the time of 130 min, when the level of blood glucose decreased from 19.3 mM to 8.2 mM (lower than the critical value of 8.3 mM), the insulin release was stopped automatically. Without a further insulin intake, the blood glucose level increased again and reached 11.4 mM at 240 min. For the second rat (red line), the blood glucose decreased from 20.8 mM to 8.2 mM in two hours. The insulin release was also stopped automatically, and the blood glucose increased to 8.8 mM at the time of 140 min. Then, the pump was driven again automatically. Gradually, with the increase in the amount of insulin, the blood glucose reached a peak of 9.9 mM at the time of 180 min, and after that, the blood glucose began to decrease until it reached 8.2 mM at the time of 210 min.

Figure 5(i) shows the closed-loop management of diabetes with a glucose intake. Similarly, the blood glucose level of the first rat (blue line) declined from 20.5 mM to 8.2 mM (lower than the critical value of 8.3 mM), and at the time of 130 min, the insulin release was then stopped automatically. At the time of 150 min, glucose (1 g/kg) was injected intraperitoneally. Without any operation, the blood glucose level increased gradually and reached 14.0 mM at the time of 240 min. For the second rat (red line), the blood glucose level decreased from 21.1 mM to 8.2 mM at 130 min. The insulin release was also stopped automatically. At the time of 140 min, the glucose (1 g/kg) was also injected intraperitoneally. The blood glucose level increased to 8.4 mM at the time of 150 min (higher than 8.3 mM). Then, the pump was turned on again automatically. Gradually, with the increase in the amount of insulin, the blood glucose level reached a peak of 10.5 mM at the time of 180 min. After that, the blood glucose level began to decrease until it reached the critical value (8.3 mM) at the time of 240 min. All these results demonstrate that the system could achieve an automatic closed-loop control of blood glucose in diabetic rats successfully.

Compared to other work for the closed-loop diabetes systems based on microneedles [22–25, 44] (Table S1), this work exhibit several obvious advantages. It can deliver insulin into subcutaneous tissue with a precise volume control that can differentiate a volume as low as 0.03 *μ*l. Insulin in subcutaneous tissue can be adsorbed faster without an insulin leakage. In addition, although there is only a microtube with a diameter in micrometers being inserted into the skin instead of arrays with a millimeter to centimeter size, the microtube sensing electrodes along the dermis layer and subcutaneous tissue can provide a sufficient electrode area to contact interstitial fluid for a sensitive glucose sensing. The device has a low cost and a small dimension as well.

In practical applications, a strong bandage or medical tape can be used to fix it on the skin to reduce the effect from a motion or an external force. The accuracy of the sensor is affected by a change in pH and temperature, and to improve the accuracy, the system can be further integrated with a pH or temperature sensor to calibrate the sensing results. Further, the insertion site can be covered by a waterproof membrane to prevent sweat from contacting the microtube to affect the accuracy. The system is disposable due to its low cost, and for a long-term diabetes management, the users can replace the microtube every few days to avoid the block of the microtube and the insulin infusion by fat granules, and other possible complications [45].

This work mainly focused on the closed-loop device parts (more specially, the sensing and pumping parts), since these are critical for the miniaturization and wearability of the closed-loop device. Although the developments of the PCB and the control algorithm are important as well, these are not the focus of this study. Therefore, this study used a simple designed PCB and control algorithm for operating the closed-loop device, which certainly should be improved in the future by the professional PCB and microprocessor engineers. Although this new closed-loop device is promising for clinical use, more specialized electronic chips, PCBs, and control algorithms should be designed and used eventually for the practical applications on human diabetic patients.

## 3. Conclusions

Here, we have successfully demonstrated an integrated closed-loop diabetes management system that consists of a microtube-type biosensor implanted into the subcutaneous tissue to detect glucose, an electroosmotic micropump to inject insulin, and a PCB to achieve the control algorithm. The biosensor was constructed with a two thin-film electrode configuration on the microtube, and the electroosmotic micropump was based on a low-cost nanoporous polycarbonate membrane. In vivo experiments proved that the system could perform a closed-loop control of blood glucose in diabetic rats intelligently. Compared to other commercial closed-loop systems, the system is small, lightweight, and cost-effective. Although these results are promising, further studies are needed to redesign the PCB to equip it with a wireless function, enhance the control algorithm, and evaluate the capability of the closed-loop system for managing blood glucose in human diabetic patients. This work can open a new paradigm for developing new closed-loop diabetes devices and may provide exciting opportunities for widespread uses among diabetes patients.

## 4. Materials and Methods

### 4.1. Apparatus and Chemicals

A potentiostat CHI660e was acquired from CH Instruments, Inc. (Shanghai, China). The magnetron sputtering was obtained from Kurt J. Lesker Co., Ltd. (America). The UV Ozone Cleaner was obtained from Ossila Co., Ltd. (Sheffield, UK). The hot plate was obtained from Jingxue Scientific Instrument Co., Ltd. (Shanghai, China). The S-4800 field emission scanning electron microscope was obtained from HITACHI, Ltd. (Tokyo, Japan). The SLA550 3D printing machine was obtained from ZRapid Tech Co., Ltd. (Suzhou, China). The commercial blood glucose meter was obtained from Sinocare Inc. (Changsha, China).

The flexible microtube was obtained from Juxin Anchuang Medical Device Co., Ltd. (Shenyang, China). The polycarbonate membrane with a diameter of 2.5 cm for constructing the electroosmotic micropump was purchased from Whatman Co., Ltd. (Meterstone, UK). The 304 stainless-steel mesh was purchased from the Sheng Cong Screen Operation Department (Changzhou, China).

Sodium hydroxide was obtained from Beijing Chemical Works (Beijing, China). Sodium dihydrogen phosphate was obtained from Tianjin Fuchen Chemical Reagent Factory (Tianjin, China). Glucose oxidase (GOD) was obtained from Toyobo Ltd. (Osaka, Japan). Glucose, glutaraldehyde, dopamine hydrochloride, and streptozotocin (STZ) were obtained from Sigma-Aldrich Inc. (Beijing, China). Ferric trichloride, calcium chloride anhydrous, potassium chloride, and hydrochloric acid were obtained from Xilong Science Co., Ltd. (Shantou, China). Potassium ferricyanide was obtained from Aladdin Inc. (Beijing, China). Sodium alginate and chitosan were obtained from Sinopharm Chemical Reagent Co., Ltd. (Shanghai, China). Sulfuric acid was obtained from Beijing Institute of Chemical Reagents (Beijing, China). Nafion solution (5%) was purchased from D&B Biological Science and Technology Co. Ltd. Ascorbic acid and uric acid were purchased from Meryer Chemical Technology Co., Ltd. (Shanghai, China). The fast-acting insulin aspart and long-acting detemir injection solution were obtained from Novo Nordisk Pharmaceutical Co., Ltd. (Beijing, China).

### 4.2. Sensing Electrode Preparation

The biosensor was fabricated with an Au working electrode and an Ag/AgCl electrode The flexible microtube was first cleaned in the UV ozone for 10 minutes. To fabricate the Au electrode, a 20 nm-thick Ti layer and a 200 nm-thick Au layer were deposited on the microtube to form two electrodes. For the Ag/AgCl electrode, a 200 nm-thick Ag layer was deposited on one Au electrode and then immersed into 50 mM ferric chloride (FeCl_3_) for 10 s to form the AgCl layer. The distance between the two electrodes on both sides was about 1 mm. Then, a CV was performed on the sensing electrode in 0.1 M H_2_SO_4_ at a rate of 1 V/s from 0.2 V to 1.2 V for 10 cycles to remove the impurities from the electrode surface and activate the electrodes. Then, the microtube was immersed into a solution containing 2.5 mM FeCl_3_, 100 mM KCl, 2.5 mM K_3_Fe(CN)_6_, and 100 mM HCl for electrodepositing the PB mediator layer onto the Au working electrode. The CV scanning was conducted at a rate of 20 mV/s from −0.15 V to 0.3 V (vs. Ag/AgCl) for 8 cycles. Finally, the biosensor was immersed into a 0.1 M KCl/HCl solution to repeat the CV scanning at 50 mV/s from −0.2 V to 0.5 V (vs. Ag/AgCl) for 4 cycles to further stabilize the PB layer onto the Au electrode.

### 4.3. Enzyme Functionalization

The sensing electrodes were firstly treated using a UV Ozone Cleaner for 10 min to obtain a hydrophilic surface before immobilizing the enzymes to bring a good contact of the enzyme with the Au electrode surface. Then, the GOD solution (50 U/*μ*l) was mixed with 1% BSA solution at a volume radio of 1 : 1, and 10 *μ*l of the mixture was coated on the surface of the Au electrode. 10 *μ*l of diluted glutaraldehyde (2%) was coated on the electrode. After drying at the 4°C for 2 h, 5 *μ*l of the chitosan solution (1%, dissolved in the 2% acetic acid) was coated onto the Au electrode. After waiting for 2 h to dry the chitosan layer, the biosensor was modified with a 5 *μ*l Nafion (0.5%) solution. The sensor was then stored in a refrigerator at 4°C overnight.

### 4.4. Calibration of H_2_O_2_ and Glucose Measurement

A constant potential of 0.1 V (vs. Ag/AgCl electrode) was applied to the working electrode of the sensor by a potentiostat to obtain the sensing current signal at room temperature (about 24°C). The sensing of glucose was studied in PBS (50 mM pH 7.0) and simulated interstitial fluid. In PBS, the biosensor was immersed into 150 *μ*l PBS, and a series of 6 *μ*l of H_2_O_2_ or glucose with different concentrations were added into the solution incrementally.

To prepare the simulated interstitial fluid, alginic acid sodium salt was first slowly added to 0.1 M KCl to produce a 1.5% w/v viscous solution. The mixture was stirred at 45°C for 4 hours in the oven. After that, a series of solid glucose with different qualities were dissolved into the mixture to generate the required concentration solution. Then, 0.2 M calcium chloride (CaCl_2_) was added until the alginate solution was completely covered. Finally, the solution was covered with parafilm and left in a refrigerator at 4°C to induce complete crosslinking. During the detection process, the biosensor was stuck into the hydrogel with different glucose concentrations.

### 4.5. Selectivity Test of the Biosensor to Different Interferences

To study the interfering effects of different electroactive substances on the sensor, a series of 6 *μ*l of uric acid, ascorbic acid, dopamine, insulin, and glucose were added into PBS with a final concentration of 0.1 mM, and different volumes (100–400 *μ*l) of insulin (10 U/*μ*l) were added into PBS as well. The current-versus-time curve was recorded during the study.

### 4.6. pH, Temperature Influence, and Storage Stability Test

The effect of different pH values was tested with PBS at different pH values. The sensing response to 4 mM glucose was studied in buffer at pH 6.0, 6.5, 7.0, 7.5, or 8.0. The effect from the temperature was studied with a hot plate. The sensing response to 4 mM glucose was evaluated at different temperatures. To measure the storage stability of the biosensor, the sensing response to 4 mM glucose was studied for three times each day. The biosensor was immersed in PBS and stored in the refrigerator at 4°C during the free time.

### 4.7. Bending Time and Angle Influence

To evaluate different bending times on the sensing performance, the microtube was bent for 50, 100, 150, and 200 times at an angle of 45°, and the current responses to 4 mM glucose were measured. The sensing responses to 4 mM glucose were also measured when the microtube was bent at the angles of 30°, 45°, 60°, 75°, and 90° to study the effect of different bending angles.

### 4.8. Electroosmotic Micropump Preparation and Flow Rate Test

The electroosmotic micropump was composed of a polycarbonate membrane with a diameter of 2.5 cm and two 304 stainless-steel meshes. These meshes with a width of 1 cm and length of 2 cm were then deposited with a Ti (20 nm)/Au (200 nm) layer to construct the Au electrode. The Au electrode could increase the mesh conductivity of and prevent it from corrosion. The two meshes covered the polycarbonate membrane completely, and part of the mesh was extended outside for connecting to the external powering device. Finally, the catheter connecting to the flexible microtube was attached to the bottom of the membrane. A 3D-printed button shape chamber with a diameter of 2 cm, a height of 1 cm, and a wall thickness of 3 mm was adhered to the top of the micropump for storing insulin. The flow rate test was studied with an electronic balance (resolution 0.01 mg). Different amounts of liquids being delivered from the micropump were collected every 5 min, and the weight was measured by the balance. Then, the wight was converted to the volume according to the density of each insulin solution to calculate the flow rate.

### 4.9. Animal Experiments

All SD rats for experiments were purchased from Charles River Biotechnology Co., Ltd. (Beijing, China). The SD rats were male with a weight of 150–200 g and 8 to 10 weeks of age. All experimental procedures were approved by the Research Ethics Committee of Peking University First Hospital (Approval Number: 202061). Streptozotocin (STZ) was used to induce the rats to be diabetic. All rats were fasted for 6 to 8 h before STZ treatment, and only water was normally provided. Solid STZ was dissolved in the 50 mM sodium citrate buffer (pH 4.5), and the STZ solution was intraperitoneally injected into rats in a single dose of 65 mg/kg (according to the weight of rats). During the first day after STZ treatment, 10% sucrose water was provided to each rat, and since the second day, regular water was provided to each rat. All rats were stored in specific pathogen-free conditions, and they were maintained in individually ventilated cages during the intermission period of the experiment. The condition had a constant temperature of 22 ± 2°C, a constant humidity of 50 ± 10%, and a 12 h light/dark cycle. The rats had access to standard laboratory food and water during free time. On the tenth day, all rats were fasted for 6–8 h, and the blood glucose level from a tail vein sample of each rat was tested to check hyperglycemia. If the value was higher than 8.3 mM, the rat was considered to be diabetic.

Before the experiments, to test the performance of the closed-loop system, all diabetic rats were fasted for 8 h and were then anesthetized with the assistance of a small animal anesthesia machine during experiments. The hair of the rat's back in the insertion site was shaved and cleaned. As the next step, the system was stuck to the rat's skin and waited for ~30 minutes for the biosensor to warm up and get stabilized in the subcutaneous before experiments. The insulin aspart Injection solution (10 U/ml) powered by an electroosmotic micropump under a 5 V DC voltage was subcutaneously injected into the rat through the microtube. To measure the change in the rat's blood glucose level at different times, the blood sample was collected from the rat's tail vein and was measured by the commercial glucose meter.

## Data Availability

The data used to support the findings of this study are included within the article and the supplementary information file.

## Supplementary Materials

Supplementary Materials Figure S1. (a) A camera image of the whole system. (b) A camera image of the system applied to the human body. Figure S2. Illustration of the working principle and control algorithm of the closed-loop system. Figure S3. Circuit diagrams of the PCB for the biosensor and micropump Figure S4. SEM image and EDS mapping analysis of the Au electrode after the immobilization of GOD and the Nafion deposition Figure S5. EDS point analysis of the Au electrode and the Ag/AgCl electrode. (a) The original Au electrode; (b) The Ag/AgCl electrode. (c) The Au electrode after the deposition of Prussian blue; (d) The Au electrode after the immobilization of GOD and the Nafion deposition. Figure S6. Electrochemical impedance spectroscopy (EIS) analysis of the biosensor before and after Prussian blue deposition. (a) Nyquist plot; (b) Bode plot. Figure S7. Performance of the microtube biosensor for detecting H2O2 in PBS. (a) Current-verses-time response upon the additions of different concentrations of H2O2. C1: 0.8 mM, C2: 2.2 mM, C3: 3 mM, C4: 6 mM, C5: 10 mM, C6: 12 mM, C7: 15 mM, C8: 16 mM, C9: 20 mM. Each error bar was three different biosensors. (b) Calibration curve of the biosensor for detecting H2O2. Figure S8. Optical images of the hematoxylin and eosin-stained pierced skin section after the insertion of microtube (a) and after applying a object (20 g) on the microtube. Figure S9. Camera images of the rat's skin after application of the microtube (with or without electrode) for 5 days. Figure S10. Camera images of the rat's skin after removing the device. Figure S11. (a) Relative error of the biosensor at different blood glucose values. (b) Distribution of the relative error. Table S1. Comparision of this work and other closed-loop diabetes management systems.
