# Supplementary material for: A Microtube-Based Wearable Closed-Loop Minisystem for Diabetes Management
Source: Research (Wash D C). 2022 Oct 25;2022:9870637. doi: 10.34133/2022/9870637 (PMC9639446; doi:10.34133/2022/9870637)
Supplement: Supplementary Materials — Figure S1. (a) A camera image of the whole system. (b) A camera image of the system applied to the human body. Figure S2. Illustration of the working principle and control algorithm of the closed-loop system. Figure S3. Circuit diagrams of the PCB for the biosensor and micropump Figure S4. SEM image and EDS mapping analysis of the Au electrode after the immobilization of GOD and the Nafion deposition Figure S5. EDS point analysis of the Au electrode and the Ag/AgCl electrode. (a) The original Au electrode; (b) The Ag/AgCl electrode. (c) The Au electrode after the deposition of Prussian blue; (d) The Au electrode after the immobilization of GOD and the Nafion deposition. Figure S6. Electrochemical impedance spectroscopy (EIS) analysis of the biosensor before and after Prussian blue deposition. (a) Nyquist plot; (b) Bode plot. Figure S7. Performance of the microtube biosensor for detecting H2O2 in PBS. (a) Current-verses-time response upon the additions of different concentrations of H2O2. C1: 0.8 mM, C2: 2.2 mM, C3: 3 mM, C4: 6 mM, C5: 10 mM, C6: 12 mM, C7: 15 mM, C8: 16 mM, C9: 20 mM. Each error bar was three different biosensors. (b) Calibration curve of the biosensor for detecting H2O2. Figure S8. Optical images of the hematoxylin and eosin-stained pierced skin section after the insertion of microtube (a) and after applying a object (20 g) on the microtube. Figure S9. Camera images of the rat's skin after application of the microtube (with or without electrode) for 5 days. Figure S10. Camera images of the rat's skin after removing the device. Figure S11. (a) Relative error of the biosensor at different blood glucose values. (b) Distribution of the relative error. Table S1. Comparision of this work and other closed-loop diabetes management systems. [file 9870637.f1.docx]

**Supplementary Information for**

**A Microtube-based Wearable Closed-Loop Minisystem for Diabetes Management**

*Yiqun Liu^1+^, Qi Yu^2+^, Xiaojin Luo^1^, Le Ye^3^, Li Yang^2*^, Yue Cui^1*^*

^1^School of Materials Science and Engineering, Peking University; First Hospital Interdisciplinary Research Center, Peking University, Beijing 10871, P.R. China

^2^Renal Division, Peking University First Hospital; Peking University Institute of Nephrology; Key Laboratory of Renal Disease, Ministry of Health of China; Key Laboratory of Chronic Kidney Disease Prevention and Treatment (Peking University), Ministry of Education, Beijing 100034, P.R. China

^3^School of Integrated Circuits, Peking University, Beijing 10871, P.R. China

*Corresponding authors

E-mail: ycui@pku.edu.cn, li.yang@bjmu.edu.cn

+These authors contribute equally to the manuscript


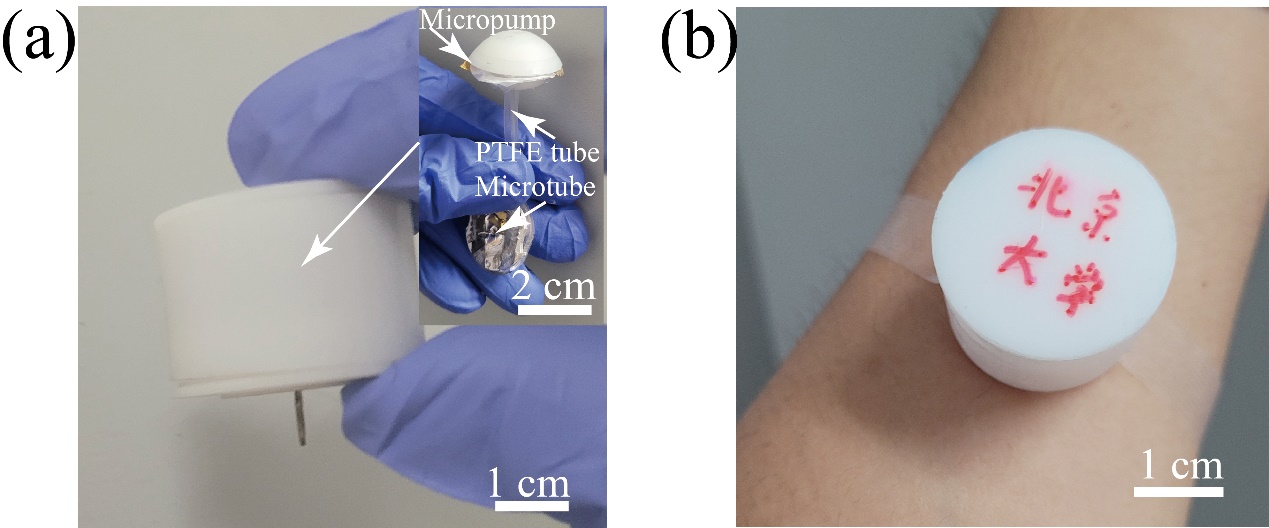


**Figure S1.** (a) A camera image of the whole system. (b) A camera image of the system applied to the human body.


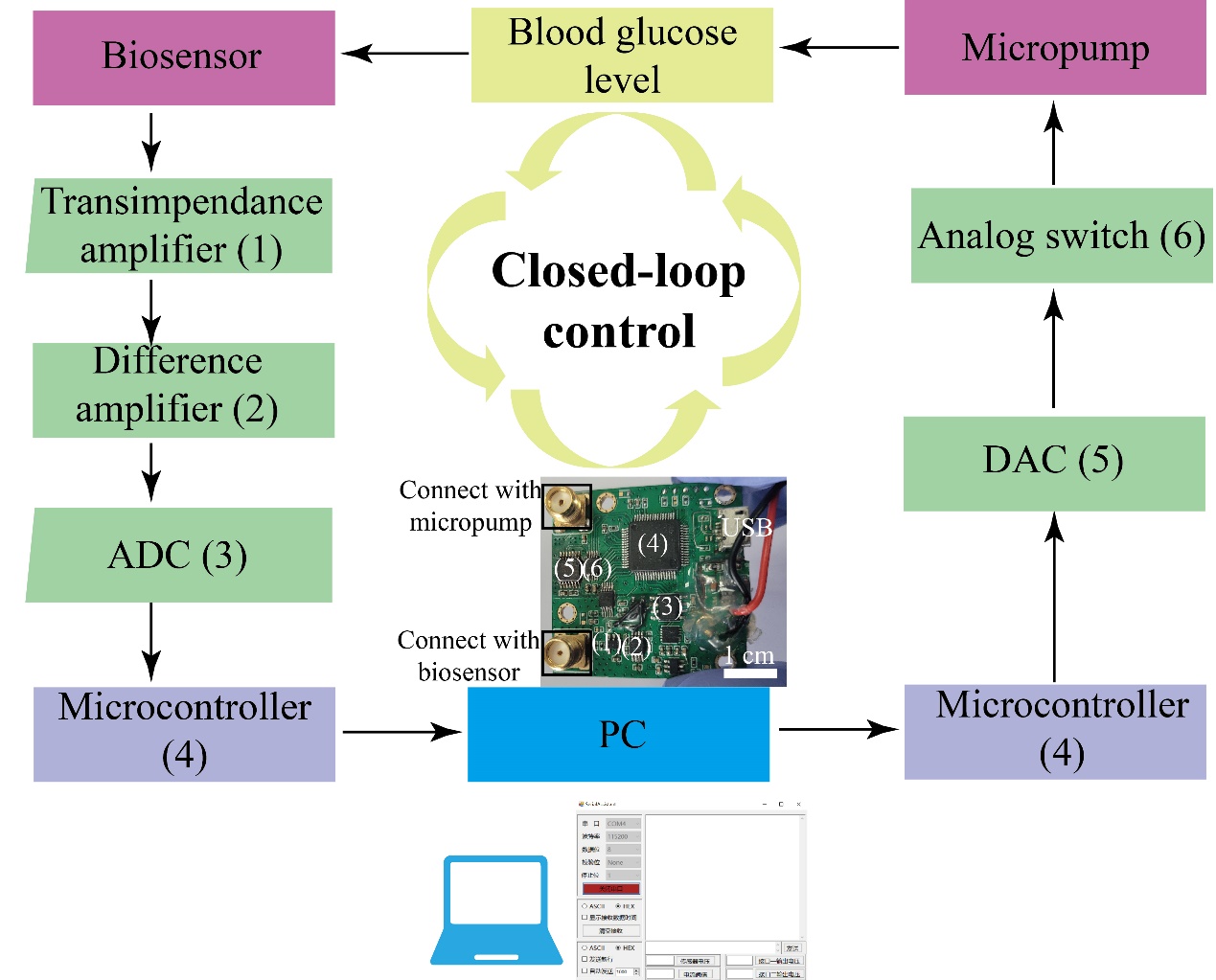


**Figure S2.** Illustration of the working principle and the control algorithm of the closed-loop system.


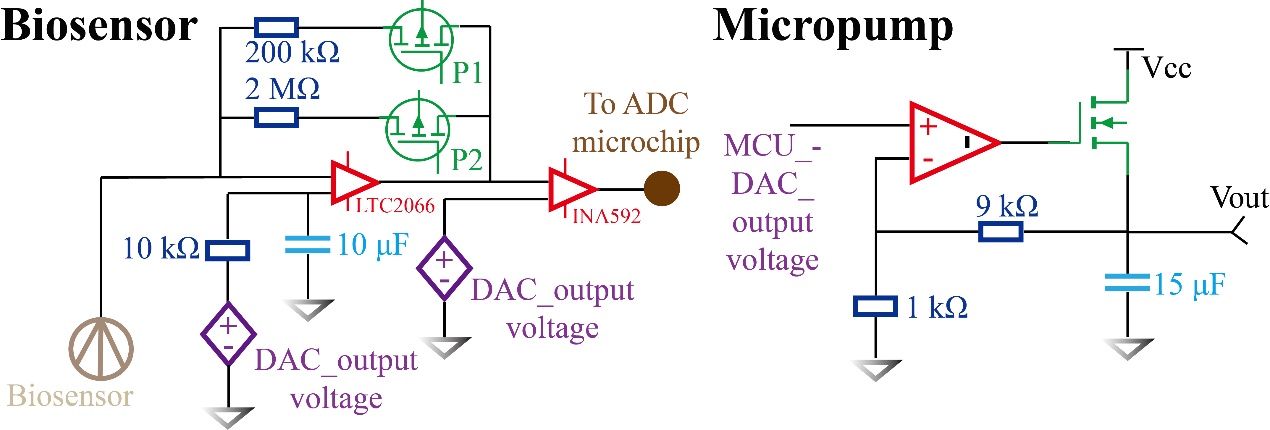


**Figure S3.** Circuit diagrams of the PCB for the biosensor and micropump


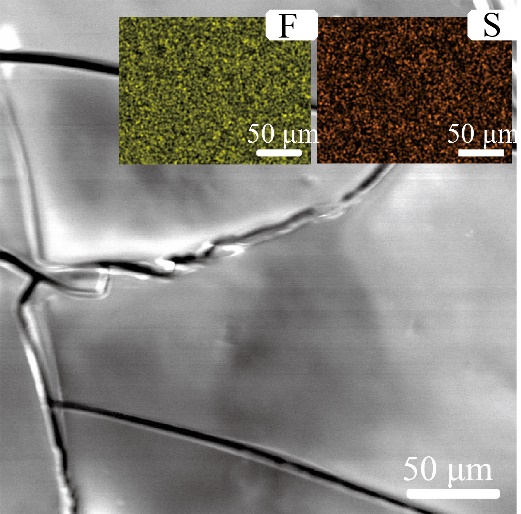


**Figure S4.** SEM image and EDS mapping analysis of the Au electrode after the immobilization of GOD and the Nafion deposition


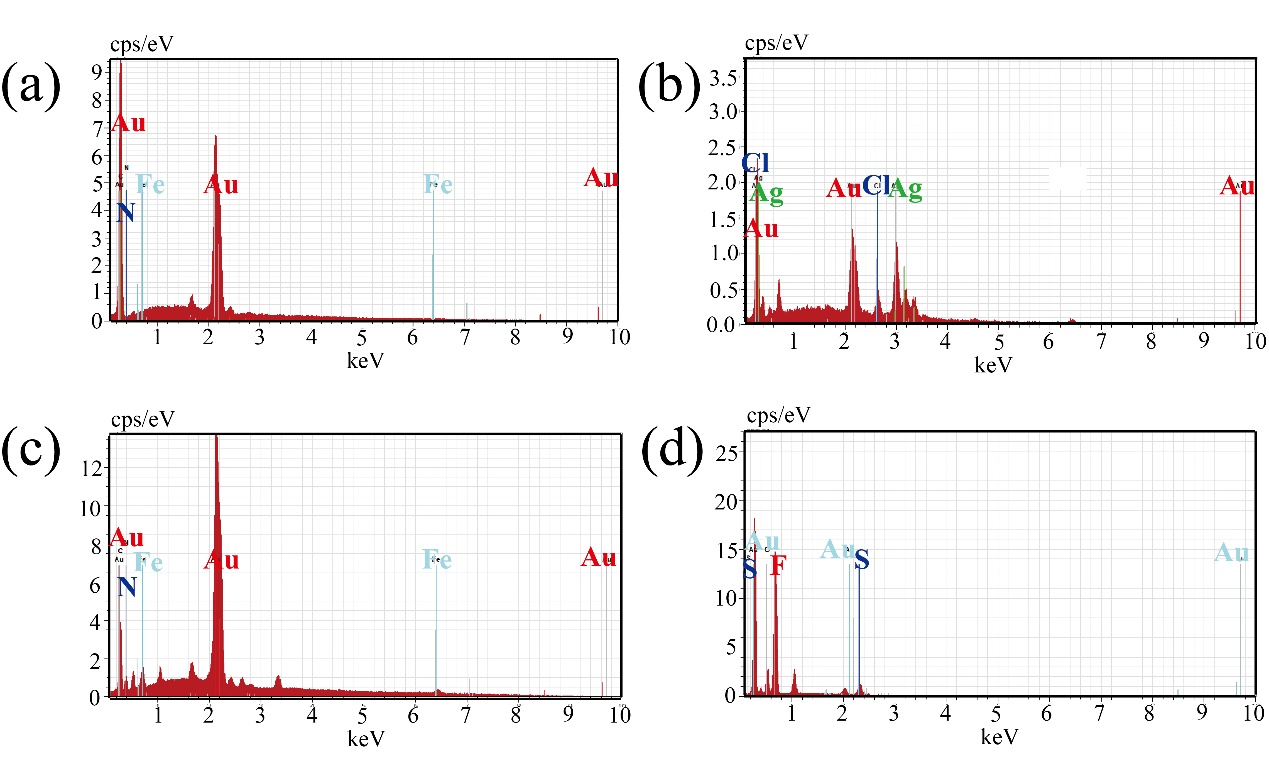


**Figure S5.** EDS point analysis of the Au electrode and the Ag/AgCl electrode. (a) The original Au electrode; (b) The Ag/AgCl electrode. (c) The Au electrode after the deposition of Prussian blue; (d) The Au electrode after the immobilization of GOD and the Nafion deposition.


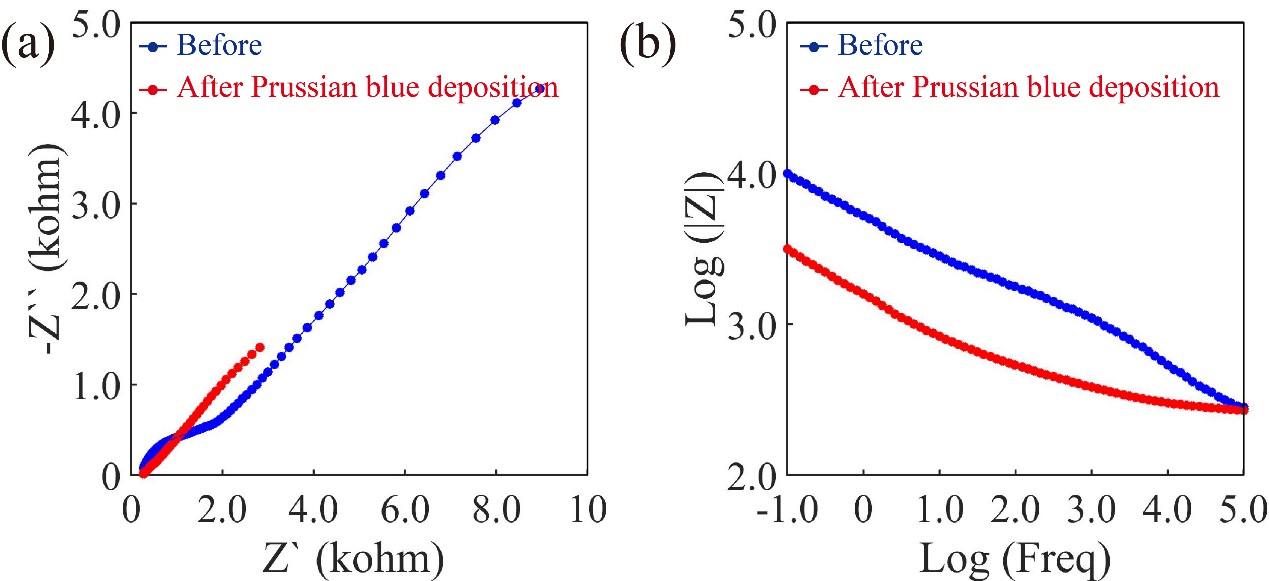


**Figure S6.** Electrochemical impedance spectroscopy (EIS) analysis of the biosensor before and after Prussian blue deposition. (a) Nyquist plot; (b) Bode plot.


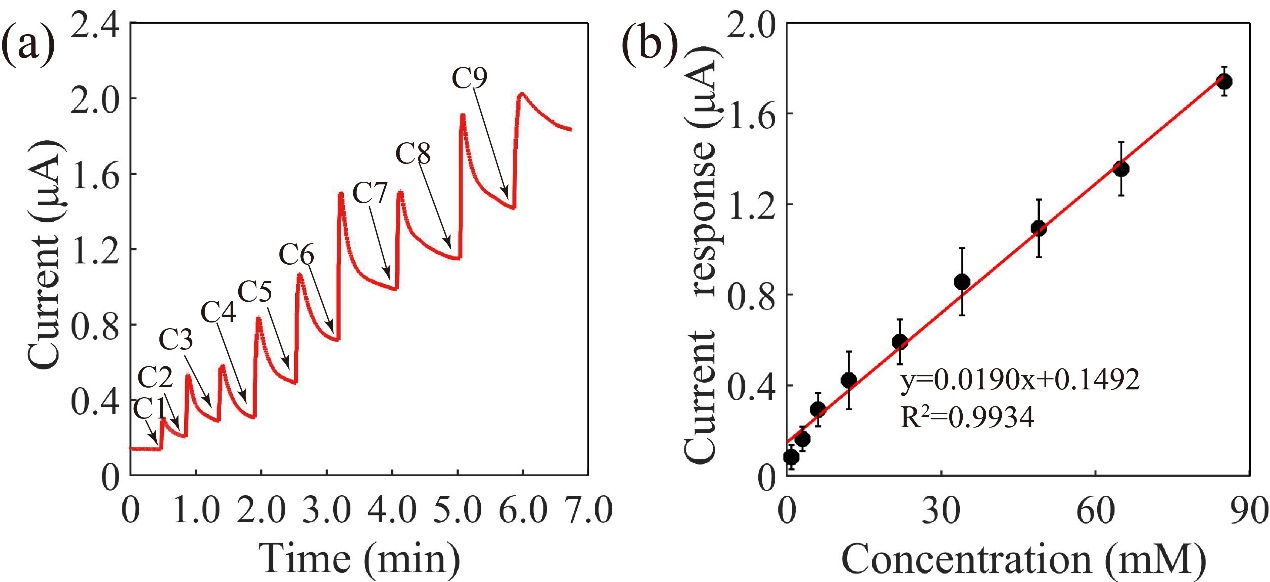


**Figure S7.** Performance of the microtube biosensor for detecting H_2_O_2_ in PBS. (a) Current-verses-time response upon the additions of different concentrations of H_2_O_2_. C1: 0.8 mM, C2: 2.2 mM, C3: 3 mM, C4: 6 mM, C5: 10 mM, C6: 12 mM, C7: 15 mM, C8: 16 mM, C9: 20 mM. Each error bar was three different biosensors. (b) Calibration curve of the biosensor for detecting H_2_O_2_.


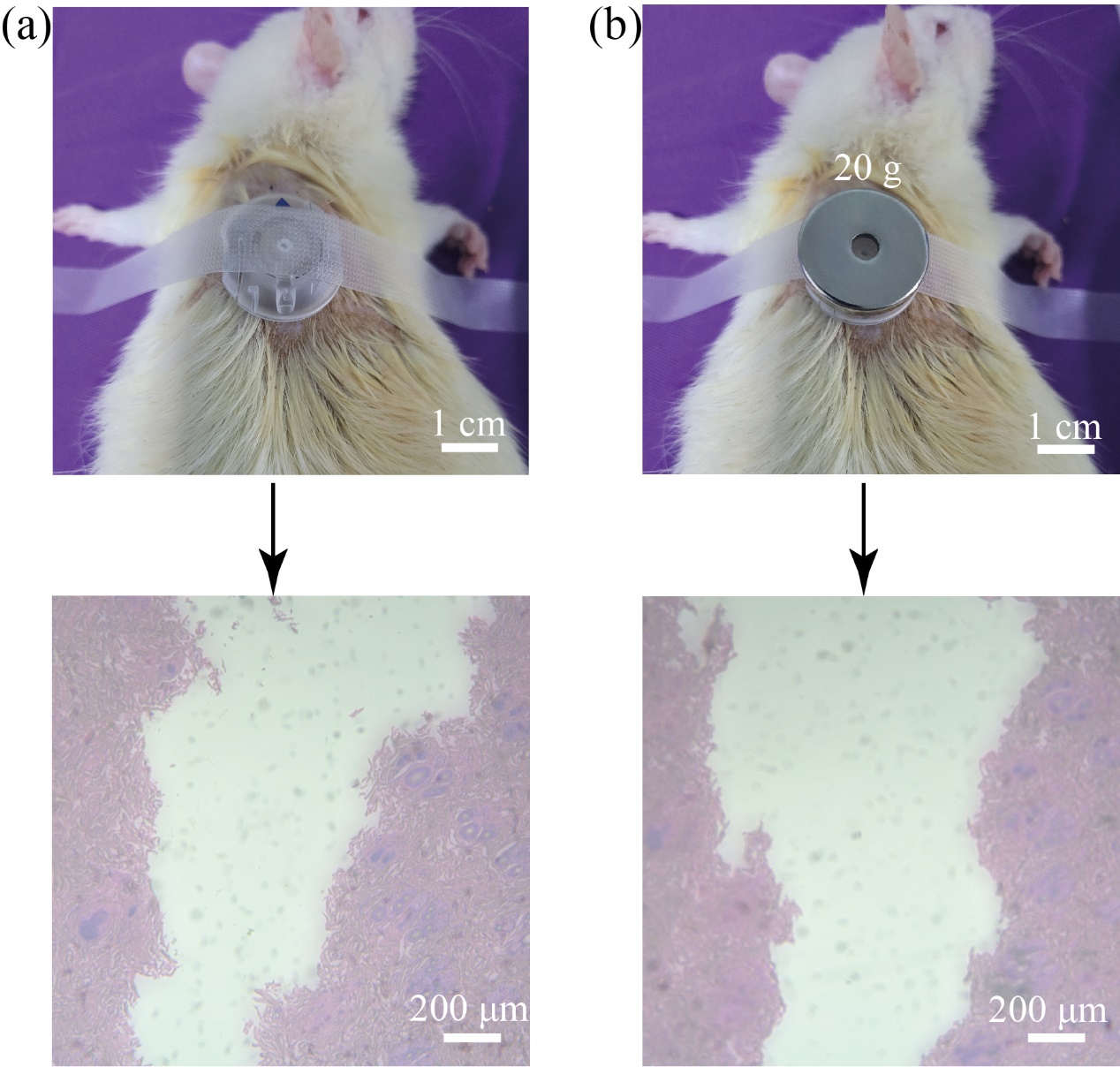


**Figure S8.** Optical images of the hematoxylin and eosin-stained pierced skin section after the insertion of microtube (a) and after applying a object (20 g) on the microtube.


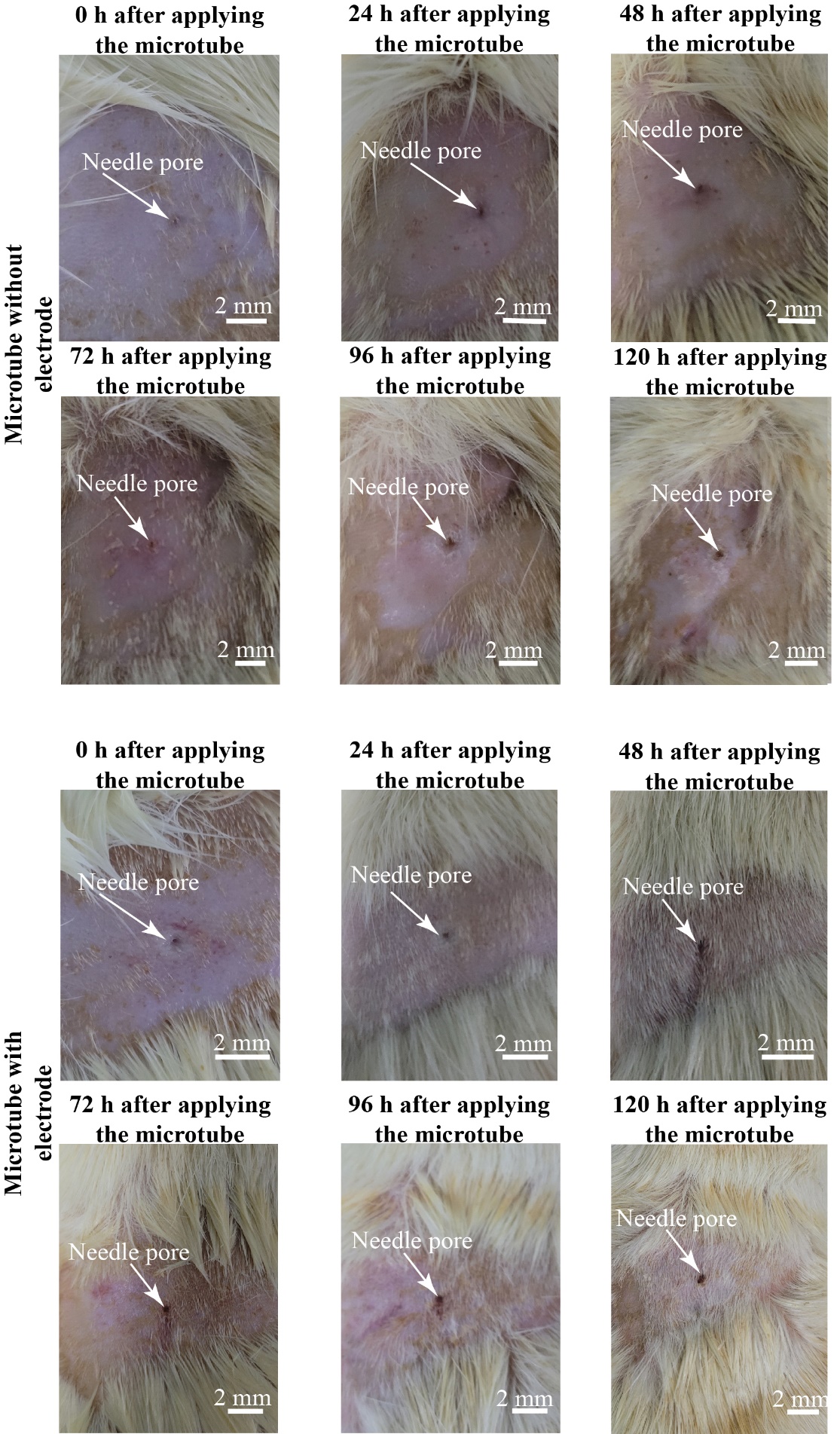


**Figure S9.** Camera images of the rat’s skin after applying the microtube (with or without electrode) for 5 days.


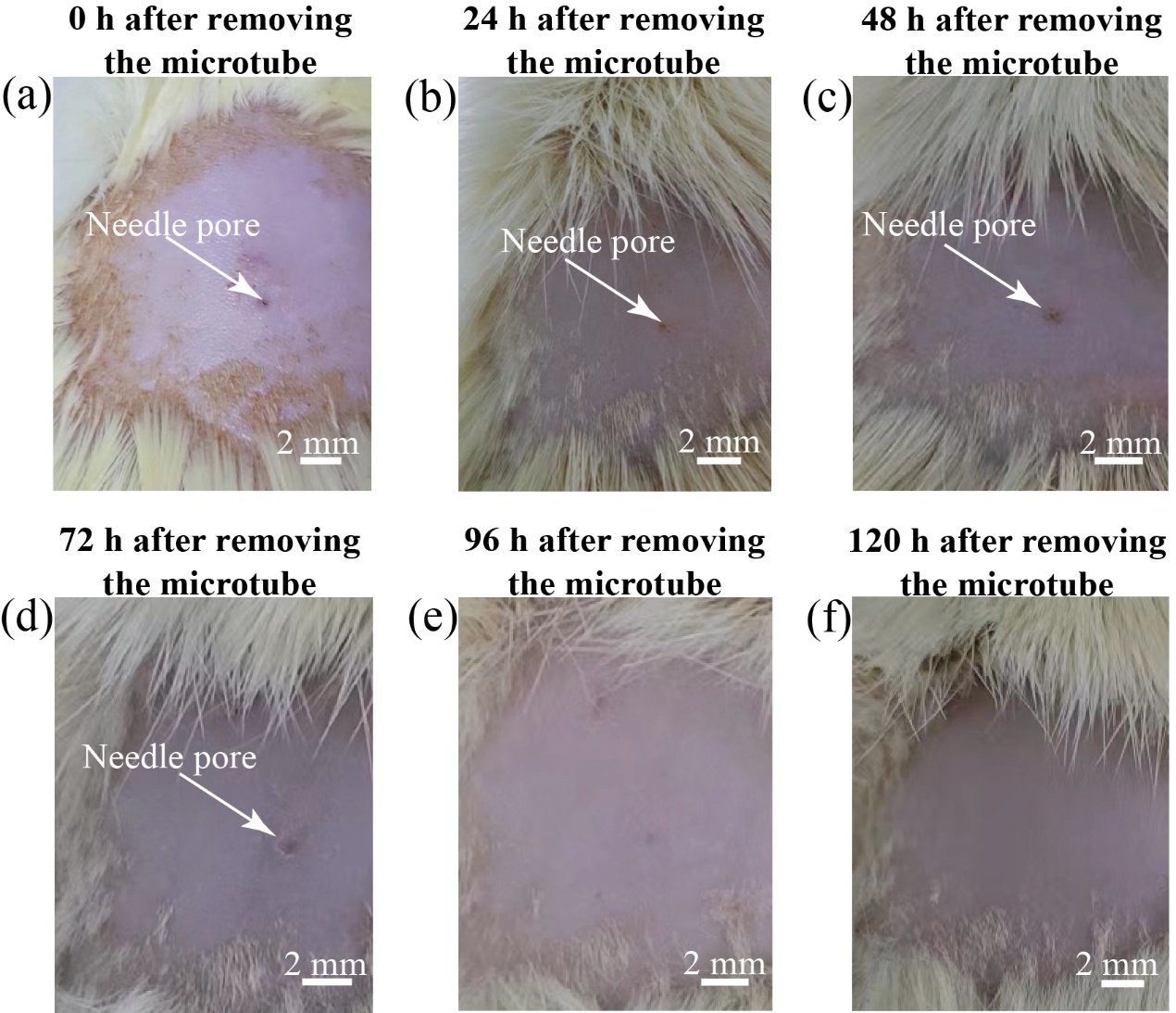


**Figure S10.** Camera images of the rat’s skin after removing the microtube. (a) 0 h after removing the microtube; (b) 24 h after removing the microtube; (c) 48 h after removing the microtube; (d) 72 h after removing the microtube; (e) 96 h after removing the microtube; (f) 120 h after removing the microtube.


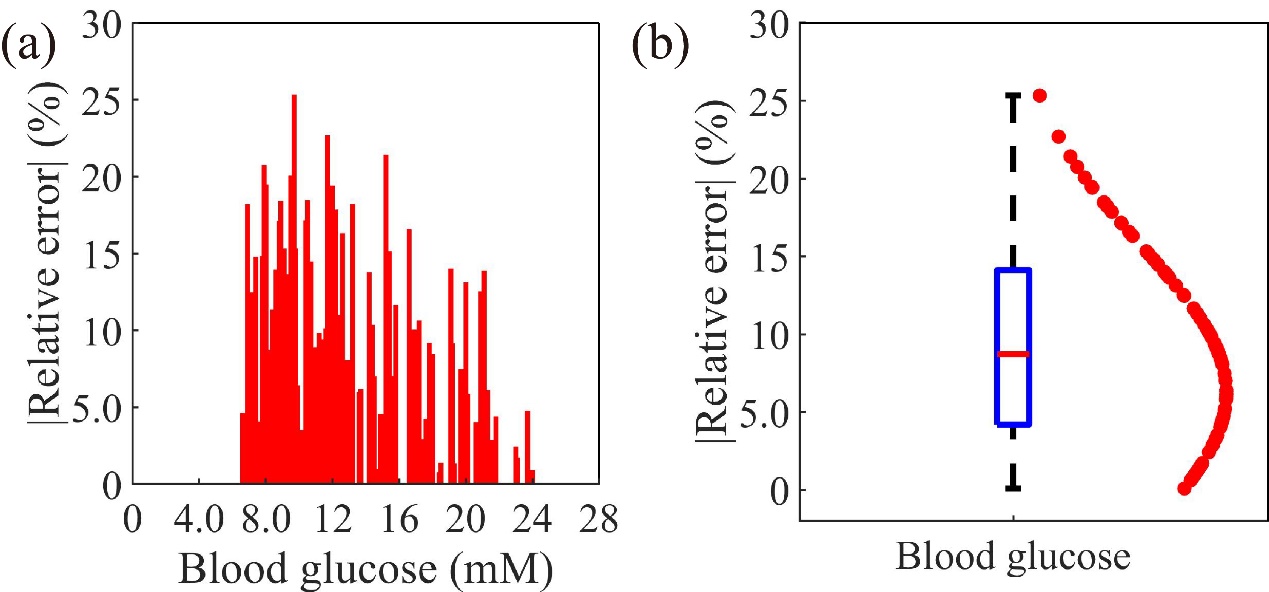


**Figure S11.** (a) Relative error of the biosensor at different blood glucose values. (b) Distribution of the relative error.

**Table S1.** Comparision of this work and other closed-loop diabetes management systems

| Device compositions | Glucose sensing layer | Glucose sensing sensitivity and range | Drugs infusion layer | Drugs release rate | Leakage of drugs | Reference |
| --- | --- | --- | --- | --- | --- | --- |
| Silicon-nanowire field-effect transistors microneedle array with a 3D printed syringe | Interstitial fluid in dermis | I/I0 increased ~125% for 10 mM glucose in buffer;  0-10 mM; | Insulin in dermis | No precise release rate | Maybe | [1] |
| Wearable patch based on a sweat biosensor and polymeric thermoresponsive microneedles | Sweat on the skin | 1.0 μA/mM in buffer;  0.01-0.7 mM; | Metformin in dermis | No precise release rate | No | [2] |
| Mesoporous microneedle array based on iontophoresis | Interstitial fluid extracted from skin | 219 nA/mM in buffer;  0-20 mM; | Insulin in dermis | ~5 IU in vivo release of 4 mg/ml for 3 h | Maybe | [3] |
| Microneedle array with electroosmotic pump | Interstitial fluid in dermis | 0.351 μA/mM in PBS;  0-30 mM; | Insulin in dermis | Maximum flow rate of 9.42 μL/min for 10 U/ml insulin | Maybe | [4] |
| Glucose-responsive insulin microneedle array | Interstitial fluid in dermis | No precise glucose sensing | Insulin in dermis | No precise release rate | No | [5] |
| Microtube with electroosmotic pump | Interstitial fluid in dermis and subcutaneous tissue | 0.0901 μA/mM in vivo;  0.8-34 mM | Insulin in subcutaneous tissue | Maximum flow rate of 3.376 μL/min for 10 U/ml insulin | No | This work |

**References**

[1] O. Heifler *et al.*, "Clinic-on-a-Needle Array toward Future Minimally Invasive Wearable Artificial Pancreas Applications," *ACS Nano*, vol. 15, no. 7, pp. 12019-12033, 2021.

[2] H. Lee *et al.*, "A graphene-based electrochemical device with thermoresponsive microneedles for diabetes monitoring and therapy," *Nat. Nanotechnol.*, vol. 11, no. 6, pp. 566-572, 2016.

[3] X. L. Li *et al.*, "A Fully Integrated Closed-Loop System Based on Mesoporous Microneedles-Iontophoresis for Diabetes Treatment," *Adv. Sci.*, vol., no., pp. 15.

[4] X. Luo *et al.*, "Closed-Loop Diabetes Minipatch Based on a Biosensor and an Electroosmotic Pump on Hollow Biodegradable Microneedles," *ACS Sens.*, vol., no., pp., 2022.

[5] J. C. Yu *et al.*, "Glucose-responsive insulin patch for the regulation of blood glucose in mice and minipigs," *Nat. Biomed. Eng*, vol. 4, no. 5, pp. 499-506, 2020.
